# Microfluidic Modules Integrated with Microwave Components—Overview of Applications from the Perspective of Different Manufacturing Technologies

**DOI:** 10.3390/s21051710

**Published:** 2021-03-02

**Authors:** Laura Jasińska, Karol Malecha

**Affiliations:** Department of Microsystems, Faculty of Microsystem Electronics and Photonics, Wrocław University of Science and Technology, 50-370 Wrocław, Poland; karol.malecha@pwr.edu.pl

**Keywords:** microfluidics, microwaves, sensors, LTCC, PDMS, PMMA, silicon, microfluidic-microwave devices

## Abstract

The constant increase in the number of microfluidic-microwave devices can be explained by various advantages, such as relatively easy integration of various microwave circuits in the device, which contains microfluidic components. To achieve the aforementioned solutions, four trends of manufacturing appear—manufacturing based on epoxy-glass laminates, polymer materials (mostly common in use are polydimethylsiloxane (PDMS) and polymethyl 2-methylpropenoate (PMMA)), glass/silicon substrates, and Low-Temperature Cofired Ceramics (LTCCs). Additionally, the domains of applications the microwave-microfluidic devices can be divided into three main fields—dielectric heating, microwave-based detection in microfluidic devices, and the reactors for microwave-enhanced chemistry. Such an approach allows heating or delivering the microwave power to the liquid in the microchannels, as well as the detection of its dielectric parameters. This article consists of a literature review of exemplary solutions that are based on the above-mentioned technologies with the possibilities, comparison, and exemplary applications based on each aforementioned technology.

## 1. Introduction

The number of publications on microfluidic devices, which describe the aspects strongly connected with microfluidics, shows that they are an increasingly researched area; based on the Scopus database, the number of the publications related to the field of microfluidics reached nearly 6000 records in 2020. This is caused by many factors, such as the minimization of the necessary samples’ volumes and, due to the low dimensions of the microfluidic components, the possibility of performing many reactions at the same time in one microsystem. Such devices are mainly called Lab on Chip (LOC) microsystems [1,2,3,4,5,6,7] and Micro Total Analysis Systems (µTAS—these microsystems allow chemical laboratory operations to be performed on a single, miniaturized device) [8].

**Figure 1:**
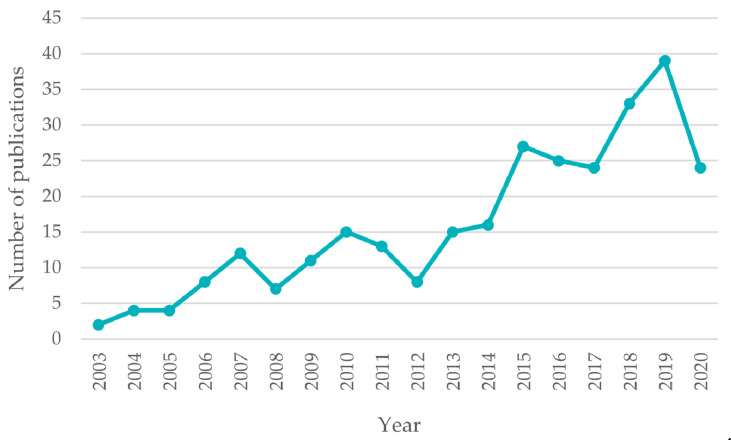
The growing number of publications on the topic of microfluidic-microwave devices. Based on the data obtained from Scopus [16].

The use of microfluidic devices with integrated microwave components is notable mainly in biochemical and chemical applications, such as detecting the biological material [9,10,11,12]. Furthermore, due to the possibility of noncontact sensing and delivering the microwaves to the systems (using the antenna-based circuits), the possibilities are still increasing [13,14,15]. This trend of microfluidic-microwave devices can be observed, e.g., in the number of publications registered in the Scopus database, which is shown in Figure 1.

Furthermore, due to the simplified geometries of the structures, the use of such systems often does not require specialized staff. Thus, these systems became common solutions in many areas, such as biotechnology, biochemistry, medical applications, etc. Moreover, the choice of the microwave methods allows different systems to be fabricated, which can be based on coaxial waveguides, as in [17], which significantly expands on the possibilities of applications.

The main goal of the recent review is to verify the state of the art of the microfluidic-microwave devices from the perspective of technologies. Due to this, the second section includes an overview of the technologies that are used to manufacture the microfluidic-microwave devices. In this section, the comparison between the substrate materials is included. Moreover, the third section describes the selected microwave devices with the integrated microfluidic component divided into three subsections. Mentioned subsections include the applications of such systems in three domains, in which the microfluidic-microwave devices are applied. The first domain is microwave spectroscopy, which allows one to observe and analyze the concentrations of the selected substances (both the organic and nonorganic ones) in real-time. The second one is the dielectric heating of the liquids, as an alternative solution for conventional heating, presented, e.g., in [18]. The third is an application of microwaves to influence the kinetics of chemical reactions, which is reflected in the growing number of microfluidic-microwave microreactors. All of the domains are described in this paper.

## 2. Substrate Materials of Microfluidic-Microwave Devices

In the literature, many materials that act as substrates for microfluidic-microwave devices can be found. However, the four most common ones are glass-epoxy laminates (especially Rogers Corp., Chandler, AZ, USA), glass/silicon wafers, various polymer materials (mostly polydimethylsiloxane, PDMS), and ceramics (Low-Temperature Cofired Ceramics, LTCC). Each of them brings different advantages and technological challenges. The electrical parameters of selected materials are shown in Table 1.

### 2.1. Glass-Epoxy Laminates

From the perspective of the microwave technique, the most characterized substrates are glass-epoxy laminates. This is related to many features, such as ease of fabricating the geometries of conductive paths and the possibility of easy integration of various active and passive electronic components [32,33,34]. Moreover, these materials are characterized by small dielectric losses and stable relative permittivity over a wide frequency range. The most common substrates dedicated to microwave purposes are those made by Rogers Corporation. However, with their adequate electrical parameters, laminates are usually less resistant to harsh environmental conditions, such as increased humidity, temperature, and various chemical agents. Moreover, the presence of the mentioned conditions is typical for microfluidic systems. Due to this, the majority of microfluidic components are made with the use of other materials and they are attached to the PCB system [33,34,35,36,37,38,39,40,41]. Due to the possibilities offered by microfluidic-microwave systems, despite some technological challenges, numerous examples of such devices can be observed in the literature.

The fabrication process of the microwave devices integrated with the microfluidic components consists of several steps. After designing the dimensions of the whole structure, the first step is typically the manufacturing of the microstrip lines (or coplanar waveguides, CPWs) and specific pattern (if necessary) of the ground plane. Most of the glass-epoxy laminates dedicated to microwave applications are covered by thin copper layers, which makes the fabrication of the conductive lines a simple process. However, the accurate integration of the microfluidic components with the circuit made on the glass-epoxy laminate is more complicated. Usually, this requires the choice of some polymer material, such as Polytetrafluoroethylene (PTFE) or PDMS, and then the integration in the designed part of the microwave microstrip circuit. Frequently, the use of some positioning components, made, e.g., by 3D printing as in [34] or some steel frame, is necessary, as shown in [42,43].

### 2.2. Glass/Silicon Substrates

One of the first microfluidic device manufactured on a silicon substrate was developed in the 1980s [44]. There are many examples of devices that are based on silicon or glass substrates operating in the microwave frequency range. This technology mostly appears in LOC microsystems, which can be made on, among other things, quartz [9,45,46,47], silicon [48], or glass [49,50] substrates. Due to fact that the material, which acts as a microwave circuits’ substrate, should be characterized by high resistivity, the silicon ones are mostly the high-resistive ones [51,52,53]. The process of manufacturing microwave devices with microfluidic components usually consists of fabricating thin-layer microwave planar waveguides on a glass or silicon substrate and adding a part constituting a microfluidic channel. Typically, the technological process consists of two stages. The first stage includes the fabricating of the conductive microwave circuit, by photolithography and/or electroplating. The second one is the manufacturing of the microfluidic components in the volume of polymer—typically PDMS—using soft lithography. Then, bonding to a glass/Si substrate containing a microwave microstrip circuit using oxygen plasma is carried out [52]. However, the possibility of omitting the plasma-bonding in the fabrication process has recently been reported, which is described well in [54].

The exemplary technological process of a device made on a glass substrate is presented in [55], where the microwave circuit was made on the glass substrate using a combination of electroplating and electrolitography. In this case, the thin layer of SiO_2_ was coated (using the magnetron sputtering) due to avoid the risk of contamination as a result of direct contact between gold microstrip lines and liquid. The fabrication of the PDMS microfluidic components was preceded by fabrication SU-8 masters on silicon wafers using soft lithography. In the last fabrication stage, the microwave glass substrate and PDMS component were treated with oxygen plasma. However, the process of manufacturing the microfluidic components is also hard to carry out, as in [56]. Due to this, the monolithic microwave-microfluidic systems are also encountered—e.g., as in works [52,57].

### 2.3. Polymers

Polymer materials are relatively standard substrates for microfluidic systems and a sufficient description of the technological processes is included in the [58]. Such materials have many advantages, such as relative ease of three-dimensional structuration and higher resistance to humidity and chemical agents in comparison to the glass-epoxy laminates. However, polymeric materials are sensitive to temperature changes and their dielectric losses significantly depend on frequency [59]. Moreover, the integration of electronic components with a single polymer substrate is more difficult in comparison to the laminates, which significantly prolongs the fabrication process. In the case described in [60], the first step of the technological process was cutting the Cyclic Olefin Copolymer (COC) substrate into a designed shape and forming the microchannel’s geometry using a CNC milling machine. Then, the slit in the microchannel was fabricated using the photolithography. In the next step, the copper self-adhesive tape was placed on the top and bottom surfaces of the COC substrate and the transmission line and ground plane were patterned.

The state of art shows a leading trend, which is the application of transparent polymers in microfluidic-microwave devices, such as PDMS, Polymethyl methacrylate, polymethyl methacrylate (PMMA) [61,62] or COC [60,63]. Typically, the polymer acts as the substrate containing the microfluidic structures made by soft photolithography. The microwave circuits are usually made of a self-adhesive copper foil [36] or using an ink-jet printing process [37]. Due to this, in comparison to the other mentioned technologies, there are only several publications that describe the microfluidic-microwave devices based only on the polymer material. The fabrication of microwave circuits is usually the last stage of fabricating the microfluidic-microwave devices based mainly on polymers.

### 2.4. Low-Temperature Cofired Ceramics

For years, LTCC (low-temperature cofired ceramics) have acted as the substrates for microwave circuits thanks to the development of substrate materials with various dielectric parameters, which are stable over a wide frequency range. Recently, LTCC have also been used successfully as a base for microfluidic modules due to the chemical resistance of LTCC, the possibility of fabrication of three-dimensional structures inside and outside the module, and humidity resistance. Due to the specificity of LTCC materials, both microwave systems (mainly antennas, but also other circuits) [64,65,66,67,68] and microfluidic modules [69,70,71,72] are fabricated as monolithic systems. The LTCC modules are the multilayer ones. Due to this, the fabrication process consists of several steps: forming the layers and the three-dimensional structures (such as microchannels and microchambers), screen printing, stacking and laminating and then, cofiring. Due to this, the LTCC technology allows manufacturing of both the conductive paths and microchannels in one technological process. Moreover, a new trend of fabricating ceramic microsystems has recently appeared—additive manufacturing, which in the near future will significantly increase the variety of fabricated ceramic microsystems [73]. Hence, LTCC seems to be suited for solutions combining microfluidic components with microwave technology, a trend that can be observed in the literature [13,74,75,76].

## 3. Areas of Microwave Applications in the Microfluidics

### 3.1. Heating

Dielectric heating uses a time-varying electric field in the microwave frequency range. This phenomenon is connected with the dipole moments, which in effect causes the dissipation of the energy as heat, which is well described in [77,78] and [79]. The use of microwave (dielectric) heating (instead of resistive heating) allows dissipation of the microwave signal in the liquid placed in the microchannel. Such a phenomenon can be achieved by precisely designing the microwave circuit for the dielectric parameters of the chosen liquid. Due to this, the risk of an unwanted increase in the temperature in parts of microsystems is reduced, which includes some electronic components. Microwaves that use heating are described in [51,52,80,81,82].

#### 3.1.1. Glass-Epoxy Laminates—Resonance Coupler for Efficient Heating of Fluids in the Microfluidic System

The authors of [32] present the possibility of heating fluids at different efficiencies in the microchannel without changing the input power level. The authors indicated that the presented concept allow the mentioned heating efficiency to be improved due to the use of two synchronized inputs with different phases. The described module was fabricated using Rogers RT/Duroid 5880 laminate with copper conductive paths. The microfluidic component was made of a quartz capillary, which was placed in a cavity located along the double split-ring resonator (DSRR). Chloroform was chosen as the test liquid.

This device was designed using COMSOL Multiphysics software. The experiment was carried out at a frequency of 2.861 GHz and a microwave input power of 180 mW. A 100 MHz shift of the resonance frequency indicated an increase in the temperature of chloroform to its boiling point (61.5 °C). The experiment included a two-port power supply, using a Wilkinson divider, a coupler with a phase shift of 180° connected to the circuit with a varactor and the DSSR resonator. The Wilkinson’s divider in the described case was used to divide microwave power. Due to this, half of the power from port 1 propagated to port 2 of the DSSR resonator. The second half of the power, after powering the circuit with the varactor located behind the coupling (with phase shift equal to 180°), was propagated to port 3, connected with the DSSR resonator. This solution, as the authors describe, allowed the substance placed in the quartz capillary to be heated by half of the input power (90 mW) with the same temperature increase effect.

#### 3.1.2. Silicon/Glass—Coplanar Waveguide (CPW)-Based Heating Device

**Figure 2:**
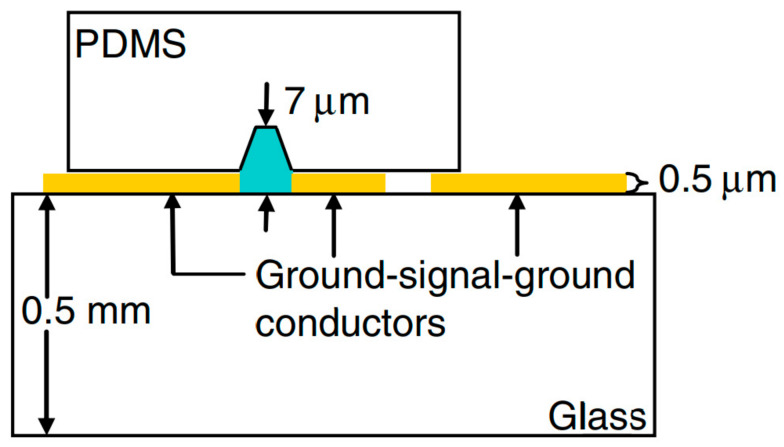
The scheme of the proposed Coplanar Waveguide (CPW) module for heating the liquids [81]. Reprinted from Shah, J.J.; Sundaresan, S.G.; Geist, J.; Reyes, D.R.; Booth, J.C.; Rao, M.V.; Gaitan, M. Microwave dielectric heating of fluids in an integrated microfluidic device. *J. Micromechanics Microengineering* **2007**, *17*, 2224–2230, doi:10.1088/0960-1317/17/11/008 with permission from IOP Publishing Ltd.

One of the methods to heat of liquids via microwaves involves the use of a circuit consisting of a CPW, described in [81]. A CPW is a type of planar waveguide, where the ground plane is located on both sides of the signal line at a certain distance. In this work, the thin-layer microwave module on the Corning Pyrex 7740 substrate was connected to the microfluidic channel made in the PDMS substrate by soft lithography as shown in Figure 2. Due to the placement of the PDMS channel between the transmission line and the ground, the effect of increasing temperature by dielectric heating was achieved with the simplified geometry of the whole device.

**Figure 3:**
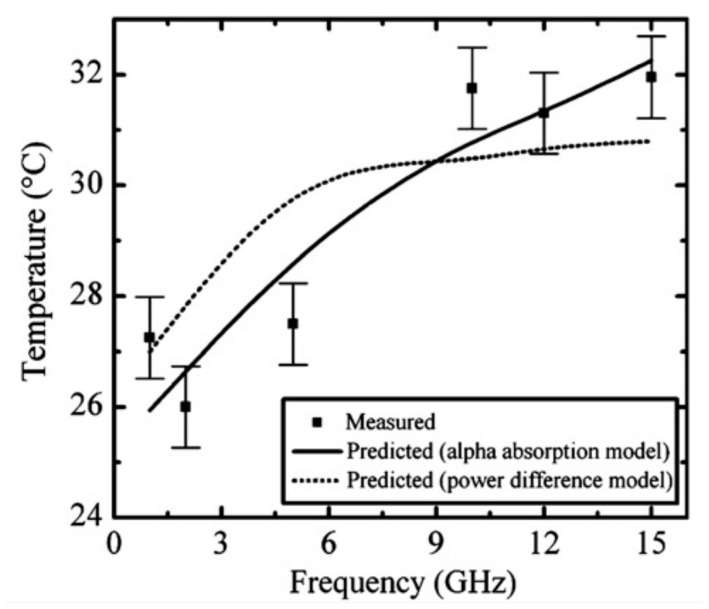
The temperature changes in the microchannel in the CPW-based module, with comparison to the predicted ones [81]. Reprinted from Shah, J.J.; Sundaresan, S.G.; Geist, J.; Reyes, D.R.; Booth, J.C.; Rao, M.V.; Gaitan, M. Microwave dielectric heating of fluids in an integrated microfluidic device. *J. Micromechanics Microengineering* **2007**, *17*, 2224–2230, doi:10.1088/0960-1317/17/11/008 with permission from IOP Publishing Ltd.

A solution of rhodamine B in bicarbonate buffer was used as the heated test liquid, and the temperature results were obtained using the approach described in [83]. The results of measurements of temperature changes as a function of frequency are shown in Figure 3.

The authors of this paper have determined the efficiency of liquid heating in a microchannel at the level of about 5% of the input power, whose changes are directly affected by the temperature of the liquid. For frequencies higher than 9 GHz, an increase in test liquid temperature to 32 °C was observed.

#### 3.1.3. Polymers—Module for Efficient Heating of a Fluid

**Figure 4:**
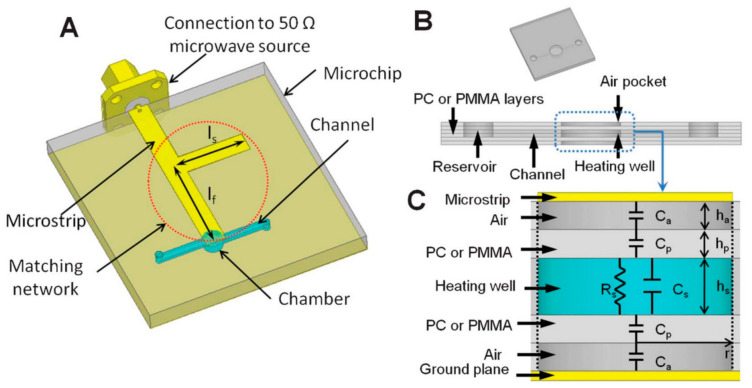
The model of Polymerase Chain Reaction (PCR) microreactor with microstrip circuit (**A**), overall and cross-section of PCR chip (**B**) and the replacement scheme of the system with the heating well as a load (**C**) [61]. Reprinted from Marchiarullo, D.J.; Sklavounos, A.H.; Oh, K.; Poe, B.L.; Barker, N.S.; Landers, J.P. Low-power microwave-mediated heating for microchip-based PCR. *Lab Chip* **2013**, *13*, 3417–3425, doi:10.1039/c3lc50461a with permission from Royal Society of Chemistry.

The use of microwaves allows the heating process of the liquid placed in the microchannel to be controlled, as well as sensing of the mentioned liquid [84]. The approach of heating using microwaves was used in the Polymerase Chain Reaction (PCR) reactors as shown in the [61]. The module was made twice initially with a Polycarbonate (PC) as a substrate and secondly, with a PMMA. The structure of the heating module is shown in Figure 4.

**Figure 5:**
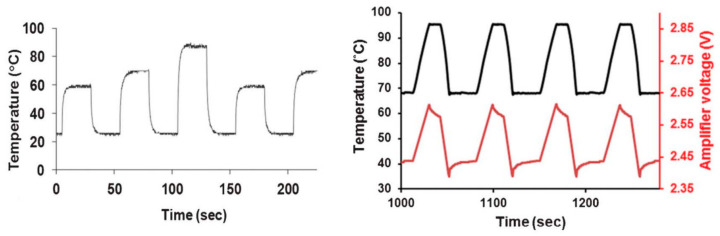
The changes of temperature in the heating well (chamber) with changing of the input signal amplitude (on left) and for various frequencies of the input voltage (on right) [61]. Reprinted from Marchiarullo, D.J.; Sklavounos, A.H.; Oh, K.; Poe, B.L.; Barker, N.S.; Landers, J.P. Low-power microwave-mediated heating for microchip-based PCR. *Lab Chip* **2013**, *13*, 3417–3425, doi:10.1039/c3lc50461a with permission from Royal Society of Chemistry.

The channel with the reaction microchamber was placed inside the structure, and the microwave circuit was cut out of self-adhesive copper tape and mounted on the surface of the polymer substrate. For the presented solution, temperature control was realized and tested in two ways. The first one assumed the change of input signal amplitude (change of microwave power value delivered to the system at a constant frequency). The second was based on the change of the signal’s frequency. The temperature changes as a function of input voltage amplitude and frequency are shown in Figure 5.

As can be seen in Figure 5, changes in the temperature of fluid with reagents were closely related to the voltage amplitude of the input signal, where the temperature increased proportionally to the applied voltage. Moreover, the temperature dependence on the frequency of the input signal showed that it is possible to carry out the PCR process for the drawn thermal cycle.

#### 3.1.4. LTCCs—Microstrip LTCC Module for Heating the Water Solutions

**Figure 6:**
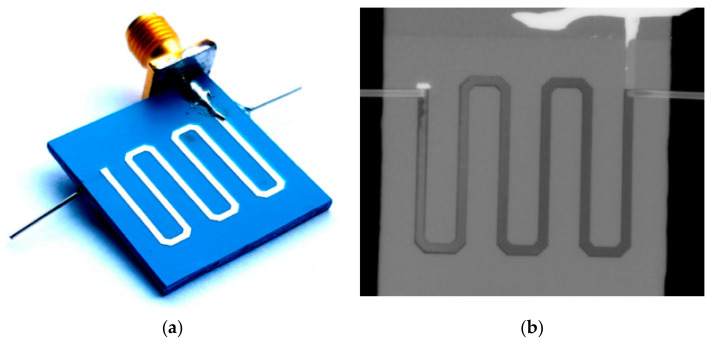
The photo of the Low-Temperature Cofired Ceramic (LTCC) device of heating (**a**) and the X-ray image with the microchannel placed below the microstrip line (**b**).

In the presented LTCC module for dielectric heating of the water solutions, the microwave circuit was designed as a microstrip line placed directly above the microchannel due to maximization of the heating effect. The device was made using eight layers of the LTCC DuPont 951 and the microwave circuit was screen-printed using the silver conductive paste ESL903A. The device and its X-ray image are shown in Figure 6.

**Figure 7:**
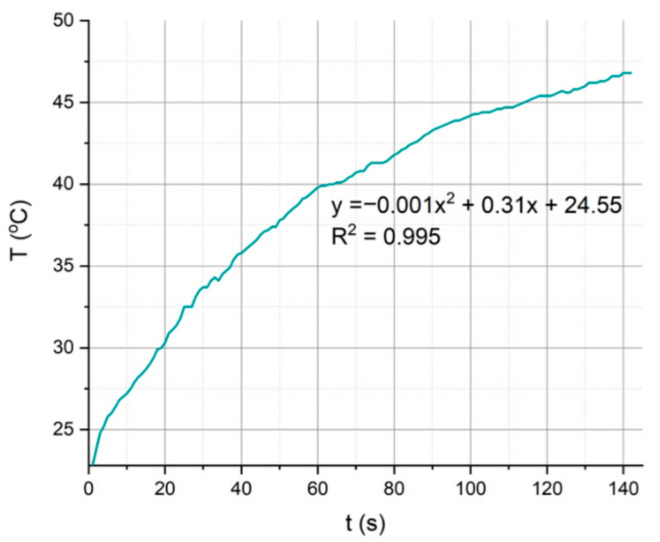
The temperature changes of the LTCC microfluidic−microwave device.

A test assessing the heating possibilities of the mentioned device was performed using the components MiniCircuits: voltage-controlled oscillator ZX95-3000W S+, attenuator −6 dB, and microwave amplifier ZHL-42. The tested liquid was delivered to the device using the peristaltic pump Ismatec Regolo ICC with a flow rate of 10 µL/min. The temperature measurements were performed using the multimeter Fluke with a thermocouple. The characteristics of temperature changes for the selected measured point are shown in Figure 7.

It can be seen that as the temperature increased, the heating efficiency decreased, which is shown in the characteristics of the graph presented above, which presents a gradually saturating curve for temperatures over 40 °C.

### 3.2. Detection

In microsystems, there are many ways to determine the composition of the tested liquids, such as optical or chemical ones. However, a relatively new trend is to use the tools that are characteristic of the microwave technique. This approach has many advantages, such as the possibility of carrying out the nondestructive tests of the solution and the characterization almost in real-time whilst being able to observe even slight changes in dielectric parameters of the tested material [85,86]. The microwave detection methods can be divided into two groups—the resonant and the nonresonant ones [51,87,88,89]. The exemplary applications of microwave circuits as a detection include the use of the resonance or differential circuits’ responses to characterize the liquid in the microchannel/microchamber [2,90,91,92,93,94,95].

#### 3.2.1. Glass-Epoxy Laminates—The Sensors for Determining the Glucose Concentration in Water

The authors of [34] present a PCB-based microfluidic-microwave sensor for determining glucose concentration in water. A prototype of this system was made on a laminate Rogers RO6002. The microwave circuit in the form of a microstrip line was made of copper. In this case, the microfluidic part was made of a polydimethylsiloxane (PDMS) microchannel and Teflon^®^ tubes, which formed the inlet and outlet of the mentioned microchannel. The construction of the presented device was based on the LC resonator (CELC, complementary LC resonator) in an odd operating mode. For a channel filled with water, the resonance frequency was equal to 1.16 GHz. As the test liquid, the solution of different concentrations of glucose in deionized water was chosen. The tests were performed in stationary, no-flow mode.

The increase in glucose concentration was accompanied by a shift in the resonance frequency with a significant increase in the amplitude of the S_21_ curve at this frequency point. The frequency shift for the test solution is described by the relation ∆𝑓𝑟 = 2.11𝜌, where 𝜌 is the glucose concentration in deionized water in mg/mL and ∆𝑓𝑟 is the resonance frequency shift expressed in MHz. Among others, a similar construction was presented in [35], where the sensor was based on a resonance circuit made on a Rogers 5880 laminate substrate and the material used for microchannels was also PTFE.

**Figure 8:**
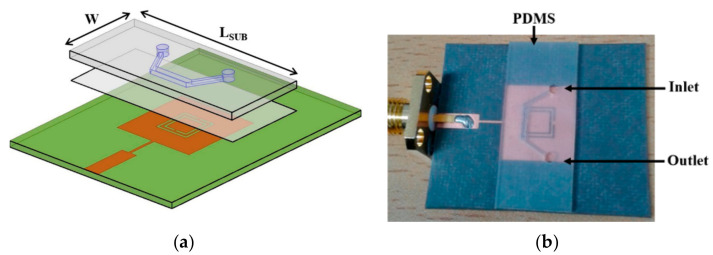
The view of the Rogers substrate (green area) with the polydimethylsiloxane (PDMS) microchannel (**a**) and the photo of the fabricated microfluidic-microwave device (**b**) [97].

Another example of a microfluidic-microwave device, where the microwave circuit was made on the glass-epoxy laminate, is the module presented in [96]. The microstrip circuit was made using the Rogers RT-5880 substrate and the microfluidic channel was fabricated in the PDMS. The sensing structure was a complementary sprit ring resonator (CSRR) and the PDMS microfluidic component was placed directly above the resonator, due to maximization of the sensing capabilities of the device. In this device, the parameter which allowed them to observe the glucose concentration in the water solutions was resonant frequency. The changes in the resonant frequency of the resonator were strongly related to the relative permittivity of the tested liquid and it was connected directly with the concentration of glucose. The authors indicated that the lower concentration of glucose that is possible to detect was 20 mg/mL [96]. The research, which was based on a similar approach described well in [97], aimed to determine the concentration of ethanol in the water solutions. The geometry of the microwave part of the device is based on the Rogers RT/Duroid 5870 substrate and was designed as a CSSR resonator. The microfluidic channel was placed above the area with a resonator, in the way shown in Figure 8.

**Figure 9:**
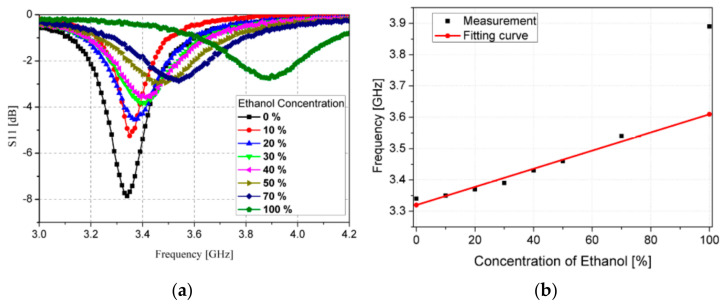
The measured reflectance S11 for the various concentration of ethanol in the deionized water (**a**) and the changing of the resonant frequency for different ethanol-deionized water solutions (**b**) [97].

After examining the repeatability of the sensor, the authors obtained the response of the mentioned device for the different concentrations of ethanol in the deionized water, which is shown in Figure 9.

In the proposed sensor, the lowest possible volume of test liquid was 3 µL, and the lowest concentration of ethanol in the ethanol–water solutions was 10%.

**Figure 10:**
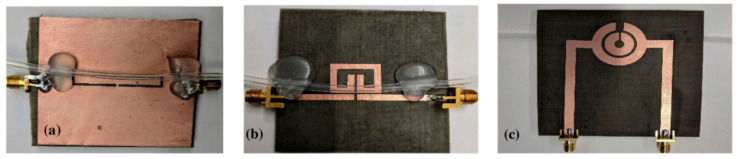
The geometries of the proposed three sensors, based on the complementary sprit ring resonator (CSRR) (**a**), extended gap SRR (EX-SRR) (**b**), and circular SRR (**c**) [98]. Reprinted from Sharafadinzadeh, N.; Abdolrazzaghi, M.; Daneshmand, M. Investigation on planar microwave sensors with enhanced sensitivity from microfluidic integration. *Sensors Actuators, A Phys.* **2020**, *301*, 111752, doi:10.1016/j.sna.2019.111752 with permission from Elsevier.

The sensors, which were fabricated using the glass-epoxy laminates and PDMS microfluidic channels, are also described in [98]. The authors designed and manufactured three resonant sensors based on CSRR, SRR with an extended gap (EX-SRR), and circular SSR. All of them were manufactured using the Rogers 5880 substrates. In the first case, the CSRR resonator was etched from the copper ground with a transmission line placed onto the substrate. The EX-SRR-based device was made directly on the Rogers substrate with the solid ground plane. The last one, circular SRR, was made in two different ways—with the PDMS microchannel placed directly on the Rogers substrate and with the microchannel embedded inside the substrate. In the second case, the microchannel was made using the PTFE tube. The geometries of the proposed sensors are shown in Figure 10.

**Figure 11:**
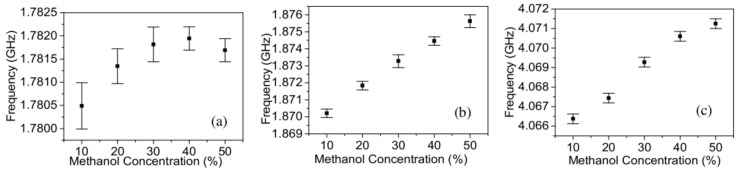
The results, which show the limit of detection for the proposed sensors, based on the CSRR (**a**), EX-SRR (**b**) and Circular SRR (**c**) [98]. Reprinted from Sharafadinzadeh, N.; Abdolrazzaghi, M.; Daneshmand, M. Investigation on planar microwave sensors with enhanced sensitivity from microfluidic integration. *Sensors Actuators, A Phys.* **2020**, *301*, 111752, doi:10.1016/j.sna.2019.111752 with permission from Elsevier.

Thus, the measurements were performed in two steps. The first step consisted of testing three sensors with the PDMS channel (not embedded in the substrate). The second one was performed for the circular SRR-based device with the Teflon tube (named carved SSR). Figure 11 shows the results, which show the detection limits of the methanol concentration in deionized water.

#### 3.2.2. The Devices Based on Glass/Silicon Substrates

**Figure 12:**
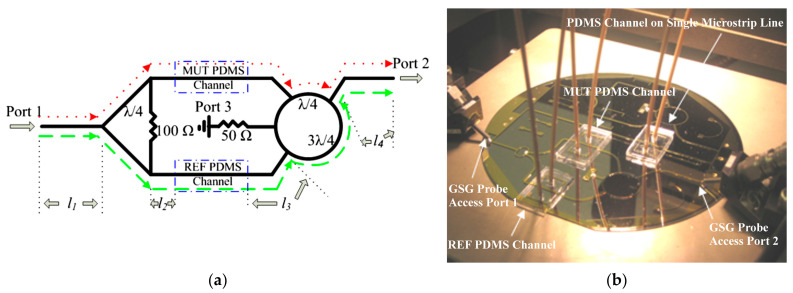
The microwave circuit scheme (**a**) and the photo (**b**) of the device for determining the concentration of the methanol/ethanol in the deionized water [49]. Reprinted from Song, C.; Wang, P.A; radio frequency device for measurement of minute dielectric property changes in microfluidic channels. *Appl. Phys. Lett.* **2009**, *94*, 1–4, doi:10.1063/1.3072806 with permission from AIP Publishing.

An exemplary microwave-microfluidic device made on a glass substrate (borosilicate Corning Pyrex 7740) was presented in [49]. The described device was designed for determining the changes of dielectric parameters of tested liquid in real-time. In this case, the microstrip lines were made of gold which was deposited on a glass substrate. The microchannel was made in a PDMS substrate. Figure 12 shows a microwave circuit of the module and a photo of the fabricated device.

**Figure 13:**
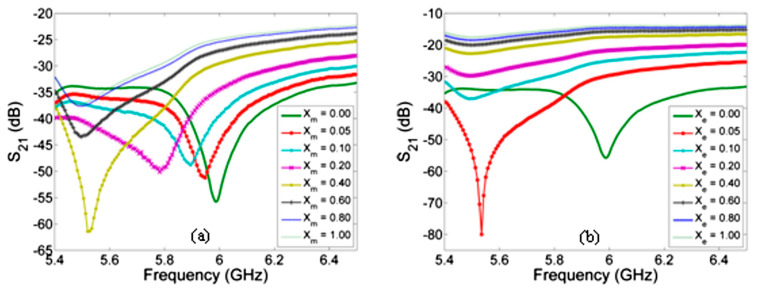
Transmittance changes for different content of methanol (**a**) and ethanol (**b**) in the deionized water in the tested solutions [49]. Reprinted from Song, C.; Wang, P. A radio frequency device for measurement of minute dielectric property changes in microfluidic channels. *Appl. Phys. Lett.* **2009**, *94*, 1–4, doi:10.1063/1.3072806 with permission from AIP Publishing.

The sensor’s operation was based on the difference in dielectric parameters of liquids placed in reference (reference material, REF) and test (material under test, MUT) channels. This module was designed to reduce background noise and 6 GHz was chosen as the operational frequency. The signal was divided using the Wilkinson divider into two branches and then propagated across two symmetrical microstrip lines. Those microstrip lines were integrated with two channels—the channel dedicated to the fluid under test and the reference one. Both lines were connected with the rat-race hybrid that acted as a 180° phase shifter. Due to the mentioned phase shift, when the dielectric parameters of the tested liquid and the reference one were the same, the phase shift ensured competition for signal cancelling on the output port. Thus, the greater the difference in liquid parameters, the stronger the signal was observed at the output port (port 2). In the manufactured system, the characteristic frequency was finally equal to 5.925 GHz. As test fluids, solutions of ethanol and methanol at different concentrations in deionized water were selected. For both fluids, the reference channel was filled with deionized water. The results for different concentrations of tested liquids are shown in Figure 13.

As the concentration of given alcohol in the solution placed in the test channel increases, an increase in the power transmitted to the system output gate for both solutions can be observed. Another device, which was designed for determining the molar fraction of the ethanol in the ethanol–water solutions, is described in [95].

**Figure 14:**
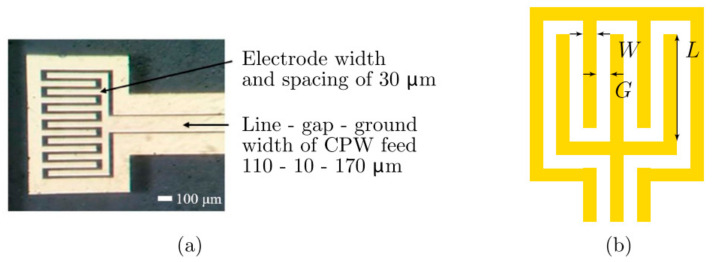
The manufactured interdigital capacitor (IDC)-based sensor (**a**) and the schematic overview, which shows the circuit’s dimensions (**b**) [99]. Reprinted from Maenhout, G.; Markovic, T.; Bao, J.; Stefanidis, G.; Ocket, I.; Nauwelaers, B. Dielectric-based temperature sensing of nanoliter water samples with a post-processing tuned matching network. *Meas. Sci. Technol.* **2020**, *31*, doi:10.1088/1361-6501/ab9937 with permission from IOP Publishing Ltd.

One of the significant sensing solutions is the temperature-changing sensor, described briefly in [99]. In this case, quartz was chosen as a substrate, and the sensor component was the interdigital capacitor (IDC), which was deposited on the substrate using UV photolithography. The microfluidic channel was fabricated using PDMS and was then bonded to the substrate by positioning the liquid in the repeatable place during the measurements. The changes in the water temperature in the proposed system can be observed by shifting the characteristic frequency. The method of mentioned temperature measurement is described in [100]. The fabricated IDC-based sensing component is shown in Figure 14.

**Figure 15:**
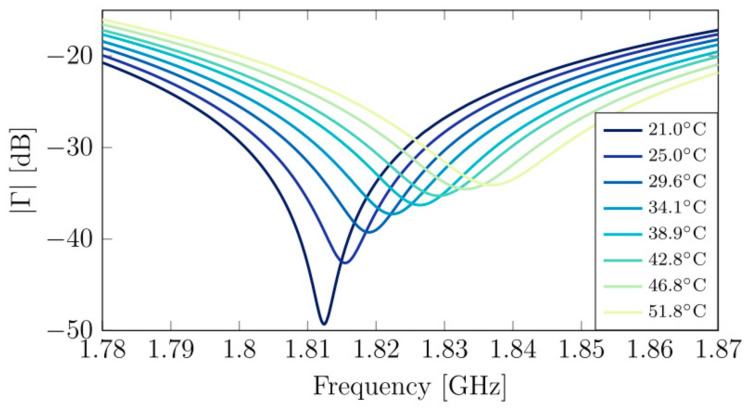
The results of reflection coefficient measurements for the different temperature water samples [99]. Reprinted from Maenhout, G.; Markovic, T.; Bao, J.; Stefanidis, G.; Ocket, I.; Nauwelaers, B. Dielectric-based temperature sensing of nanoliter water samples with a post-processing tuned matching network. *Meas. Sci. Technol.* **2020**, *31*, doi:10.1088/1361-6501/ab9937 with permission from IOP Publishing Ltd.

The measurement system consisted of three components—the lab equipment (PMA Keysight E8361A), the matching network, and the IDC with the sample under test. The observed parameter was the reflection coefficient in the frequency range of 1.78–1.87 GHz. The results are shown in Figure 15.

The accuracy for a presented solution was ±0.24 °C over a range of 20–55 °C. In [10], the authors indicated the possibility of using the IDC for sensing biological materials. Similarly, the substrate of the mentioned sensor was quartz and the components were made using microfabrication methods. In this case, SU-8 was chosen as a material of the microfluidic channel.

#### 3.2.3. Polymers—The Sensor of the Water Content in Crude Oil

Another example of a polymer microwave-microfluidic device is a sensor used to determine water content in crude oil [62]. The aforementioned sensor was made on a PMMA substrate. The microstrip circuit (T-type resonator) was applied through ink-jet printing, using ink with silver nanoparticles. In this case, the ground plane was made of self-adhesive copper foil. The microchannel was placed inside the PMMA module. The geometry of the device was developed based on the results of numerical simulations carried out using ANSYS HFSS software.

The measurements were carried out for nonwater crude oil and solution crude oil/water with 5%, 25%, and 50% volume water contents. The authors achieved the resonant frequency shift for the solutions with 5%, 25%, 50% and 100% (only water) of 55, 270, 510, and 2520 MHz, respectively. In this case, the reference frequency value was the resonant frequency of the sensor with the microchannel filled by nonwater crude oil. The results show that it is possible to determine the water content in the petroleum mixture, due to the shift of the resonance frequency towards the lower ones, with an increased volume of water in the liquid under test.

#### 3.2.4. LTCCs—Microstrip Sensor Based on Split-Ring Resonator and the Sensor for Droplet Detection

**Figure 16:**
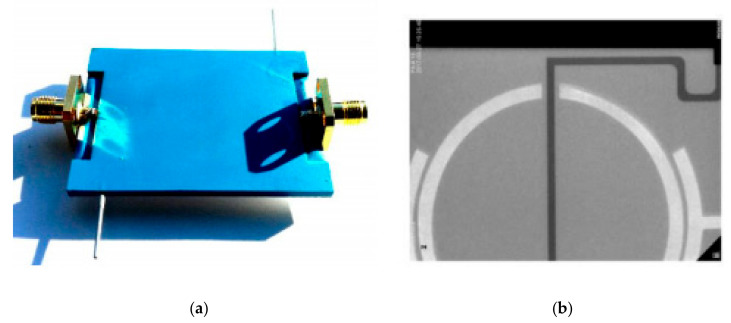
The photo of the manufactured detector (**a**) and the X-ray image with the microwave circuit of the detector (**b**) [101].

Another example is the microstrip resonator, made on DuPont 951 PX LTCC [101]. The resonator was designed with the microchannel placed in the cavities of the split-ring resonator. Such a resonator has been buried inside the LTCC structure. Due to this, changes in the dielectric parameters could be observed as a change of the resonant frequency and reflectance. The geometry of the device was designed using COMSOL Multiphysics Software and it is shown in Figure 16.

**Figure 17:**
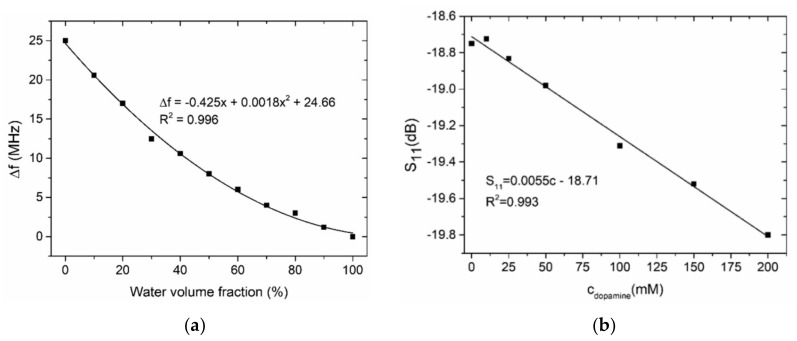
The changes of the resonance frequency for different water contents in the ethanol–water solutions (**a**) and the changes of reflectance S_11_ for various concentrations of dopamine in the buffer (**b**) [101].

In the case of the resonance sensor, the observed quantity (showing changes in the parameters of liquids in the microchannel) was reflectance S11. As a test liquid, the various concentrations of ethanol in the deionized water and the solutions with different amounts of dopamine in the phosphate-citrate buffer were chosen. The test liquids were fed to the sensor using a peristaltic pump Ismatec Regolo ICC. The obtained results for both liquid types are shown in Figure 17.

After the measurements, it was observed that changes in the volume of ethanol in deionized water, as well as changes in dopamine content, affected the response of the sensor. In [101], another device was presented. Such a module was also made using the LTCC material as a substrate, but the microwave circuit consisted of the Wilkinson divider, the symmetric transmission lines, and the rat-race hybrid, similar to the solution shown in [49].

**Figure 18:**
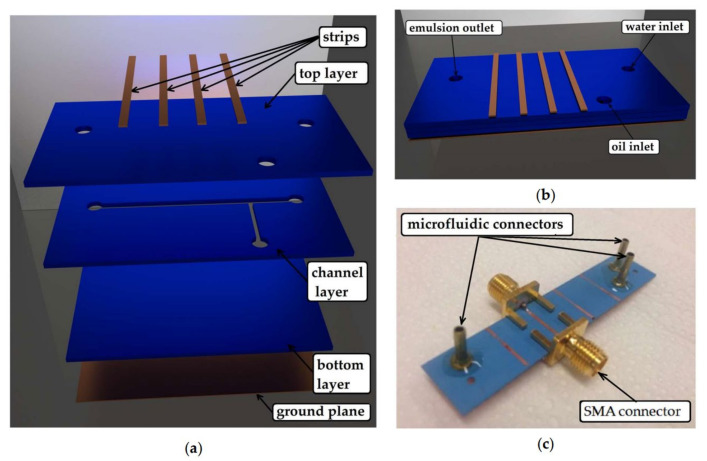
The scheme of a microfluidic-microwave device for measurements of droplets; the view of layers consisted of the module (**a**), the top view of the device (**b**), and the picture of the fabricated device (**c**) [102].

Another example is the LTCC microfluidic-microwave device for detecting the droplets in test emulsion placed in the microchannel [102]. The microwave circuit was designed to observe the presence and specific dimensions of droplets. To fabricate the emulsion, the T-junction channel was designed and fabricated, with inlets for water (continuous phase) and oil (dispersed phase). The device is shown in Figure 18.

**Figure 19:**
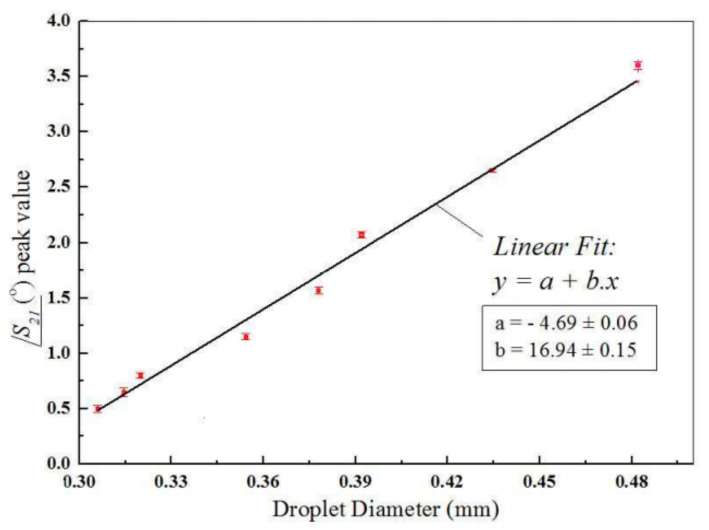
The measured peak value for different droplet diameters [102].

The measurement method consisted of observation of the phase shift of the transmittance (S21), which is the consequence of varying the relative permittivity of the emulsion in the microchannel placed below the microstrip line circuit. The authors indicated that the presence and size of a fraction of the measured droplets had an impact on the phase shift. The dependence between the dimensions of the tested droplets and the peak of the S21 parameter phase is shown in Figure 19.

The described device allows one to observe the changing parameters of the fluid (droplets) in the real-time measurements and the number of the droplets in the microchannel during the process.

**Figure 20:**
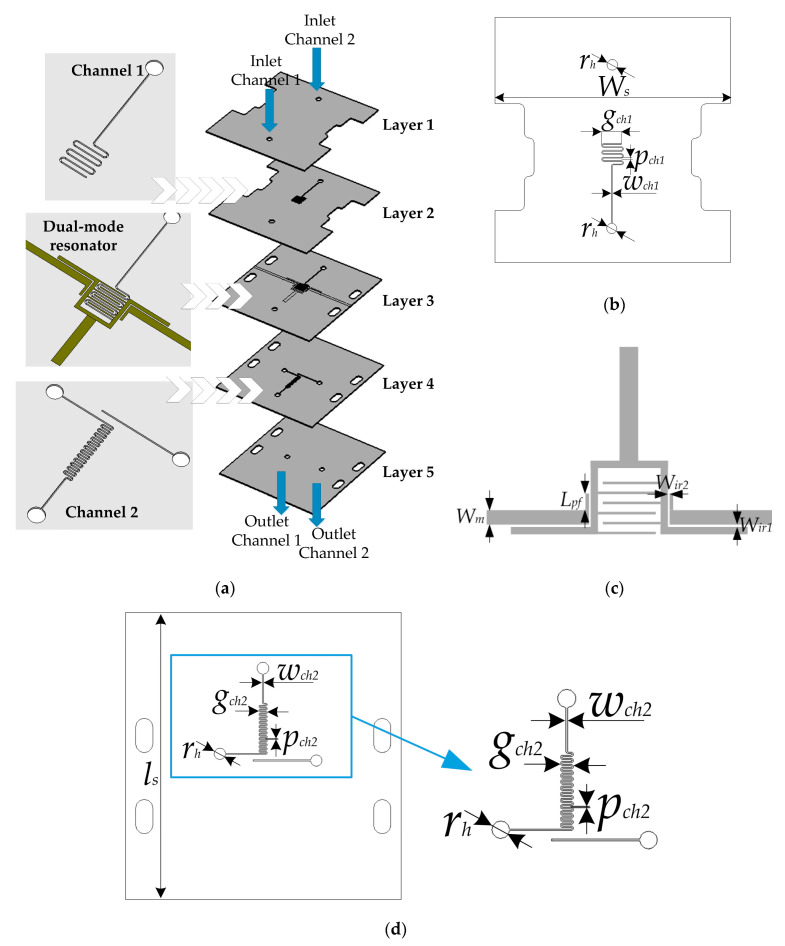
The layers’ geometries (**a**), the scheme of layer 2 with microchannel (**b**), the scheme of layer 3 with the capacitance resonator (**c**), and the scheme of layer 4 (**d**) [89].

A microwave-microfluidic sensor, which was tested with a few different liquids, is presented in [89]. The described paper describes a resonant sensor with two characteristic resonant frequencies. This approach allowed for the sensing of two fluids for each resonant frequency, which are equal to 2.02 and 3.34 GHz for the microfluidic channels filled with air. For the proposed sensor, the chosen substrate was LTCC CeramTape GC. The geometry of the proposed sensor is shown in Figure 20.

**Figure 21:**
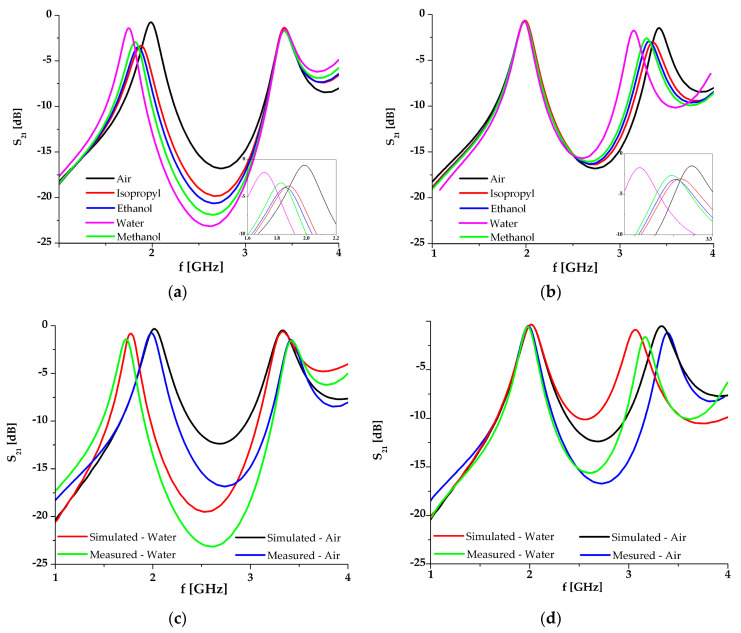
The results for the sensor with the first channel filled with tested liquids and the second one filled with air (**a**); the sensor with the first channel filled with air and the second one with the tested liquids (**b**); the comparison between the simulated and obtained results for the first channel filled by water/air (**c**) and with the second one filled with water/air (**d**) [89].

After the manufacturing process, the sensor was used to characterize the several liquids (air, isopropyl, ethanol, water and ethanol) using the VNA Agilent 8501 in the frequency range 1–4 GHz. The results are shown in Figure 21.

The authors indicated, that the first resonance was characterized by the accuracy of 3.57 MHz/ε_r_ and the second one by the accuracy of 3.91 MHz/ε_r_.

### 3.3. Impact on the Kinetics of the Chemical Reactions

Many of the changes in the chemical reactions in the solutions under microwave treatment are caused by the temperature increasing; however, nontemperature effects can also be observed [103]. One of the earliest papers, which describes this approach, is [63]. In organic chemistry, the use of microwave radiation in the chemical reactions is known as Microwave-assisted Organic Synthesis (MAOS) and Microwave-assisted Continuous Flow Organic Synthesis (MACOS) [104,105,106]. The whole aspects of the applications the microwave-assisted chemistry are described well in [107].

**Figure 22:**
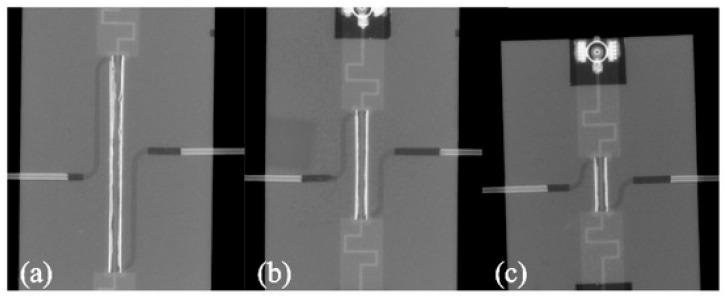
The X-ray images of the fabricated microreactors for the synthesis of the gold nanoparticles with the 20 mm (**a**), 10 mm (**b**) and 5 mm (**c**) long microreactor [108].

One of the examples of the aforementioned devices is a microwave microreactor for the synthesis of gold nanoparticles, presented in [108]. As a substrate, LTCC DP951 PX was chosen. The microwave circuit was realized as a strip line. The device was designed to effectively couple the 2.4 GHz signal. To determine the maximum power absorbed in the channel, three modules with strip lines of different lengths were made. X-ray images of the devices are shown in Figure 22.

**Figure 23:**
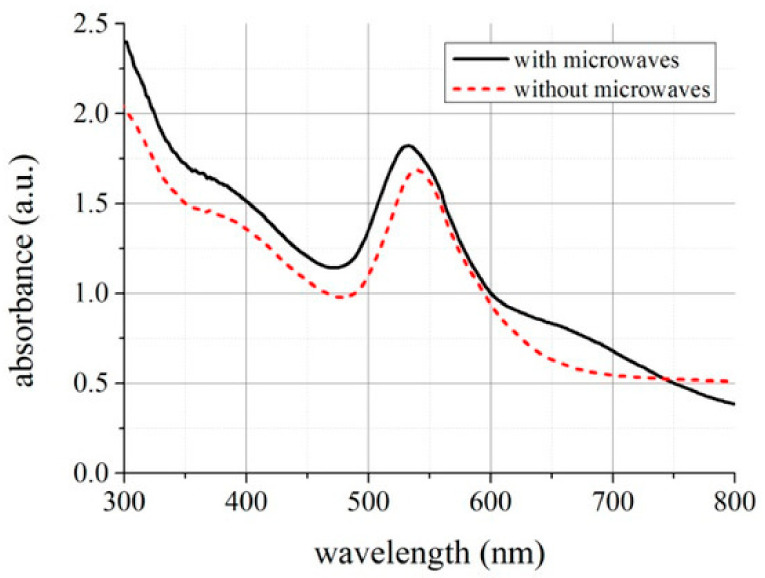
The UV–Vis spectra for the reaction mixture with and without microwaves in the microreactor [108].

Based on the measurements, it was found that about 6% of the input power was absorbed by the medium flowing through the microchannel. The authors examined the influence of microwave interaction on the process of synthesizing gold nanoparticles. The efficiency of the synthesis of gold nanoparticles was determined by UV–ViS spectra measured for the reaction mixture affected by microwaves and for the reference mixture. The obtained spectra are presented in Figure 23.

It can be observed that spectra for wavelengths equal to 524 nm are characterized by higher absorbance values for the mixture treated by the microwaves in the microreactor. The higher absorbance means a higher concentration of gold nanoparticles in the tested solution.

Another example of a microreactor for the synthesis of gold nanoparticles is a solution shown in [109]. The microreactor was designed using PTFE as a substrate, with the coaxial lines and ground planes on the bottom and top of the substrate. The microfluidic channel was designed to be placed directly between the coaxial lines inside the PTFE structure. After microwave irradiation of the solution with the reagents, the authors decided to examine the liquid by UV–Vis spectroscopy and dynamic light spectroscopy. The obtained results showed that the particle size was about 60 nm.

## 4. Comparison Between Technologies and Discussion

A comparison between the sufficient characteristics from the perspective of manufacturing the microfluidic-microwave devices is shown in Table 2.

As can be seen in Table 2, depending on the expected operating conditions and the target applications, different technologies will be appropriate.

For the glass/silicon substrates, it is possible to fabricate planar microwave circuits, as well as the microfluidic components, in a repeatable way with very high accuracy, creating a hybrid (integrated with other materials) and monolithic microfluidic-microwave devices. However, this technology carries a higher cost than ones that are fabricated on glass-epoxy laminates, polymer materials or even LTCC. Additionally, it is necessary to use a highly specialized technological line. Therefore, in cases that require a very high precision of microfluidic and microwave component geometries while maintaining high resistivity for the harsh operating conditions and the possibility of reusing the devices is an important advantage; this technology seems to be an appropriate choice, especially when the cost of creating/renting a specialized technological line is not an obstacle.

On the other hand, for polymer materials, this technology allows devices with various geometries to be produced relatively quickly, which can be useful, e.g., in fast prototyping. Moreover, when choosing the soft photolithography method, the microfluidic components can be fabricated inside the substrate, as well as on the surface. Moreover, the whole technological process is less complex than in the case of glass/silicon substrates, and the cost of the fabricating unit is also lower. However, it is impossible to obtain such accuracy of conductive components compared to the previously glass/silicon ones, which significantly limits the range of applications. Therefore, such materials seem to be suitable for fast-prototyping processes or to be integrated with the components made in different technologies, which is a common solution. Thus, polymer substrates can be appropriate for applications where the operation frequency will not change, the accuracy of the conductive paths is not the most significant aspect, corrosive chemical agents are not present, and a lower cost of the single developed device is important (especially in cases where the fabricated device will be disposable).

The glass-epoxy laminates are a good choice as their integration with polymeric microfluidic components is possible. Additionally, they are very well characterized as substrates for microwave circuits and the technological process itself has been known for several decades. Thus, in a situation where the accuracy of microfluidic components is not as significant as the accuracy of fabricating the microwave transmission lines, the whole technological process should be less costly than in the case of glass/silicon substrates and the device can be disposable—the choice of glass/epoxy laminate seems to be optimal.

In the case of LTCC, the technological process itself is more complex than in the case of polymer materials and laminates; however, it allows manufacturing of the monolithic microwave-microfluidic devices. Moreover, glass/silicon technology is less efficient and expensive compared to LTCCs; however, LTCC are used at the cost of manufacturing accuracy. Therefore, in cases when the conductive paths can be fabricated less precisely than the ones deposited on the, e.g., quartz when the positioning of the microfluidic components is significant, corrosive chemical agents and a harsh environment are present, and the device is used repeatedly, LTCC are the appropriate choice.

## 5. Conclusions

The application of microwave technology capabilities in microsystems is an area that has been used for a relatively short time. These solutions become desirable due to the relatively low cost of microwave generators and power detectors for specific frequency ranges and the possibility to observe the change of liquid parameters in real-time without the need for additional chemical reactions. Hence, it is highly probable that the solutions of microwave detectors and microreactors will constantly increase in the nearest feature.

In this review, the possibilities of manufacturing microfluidic-microwave modules are presented using various substrates: glass-epoxy laminates, polymer materials, glass/silicon substrates, and LTCC. The choice of each of these materials brings various possibilities and technological challenges. Laminates and polymeric materials require hybrid solutions to produce microfluidic-microwave devices. On the other hand, the choice of technology based on silicon and glass allows monolithic microfluidic-microwave devices to be created, which significantly broadens the range of applications. Similar possibilities are offered by LTCC, which are a less expensive solution than the use of glass/silicon substrates. However, it is extremely difficult to fabricate low-dimensional structures (for both microchannels and conductive paths). Microfluidic-microwave systems are an area that is constantly developing, and the multitude of manufacturing possibilities enables the selection of materials according to the needs and the planned unit production cost.

## Figures and Tables

**Table 1:** The selected properties of the materials that act as substrates for microfluidic or/and microwave components in the microfluidic-microwave devices. Based on [19,20,21,22,23,24,25,26,27,28,29,30,31].

Material	Silicon	Borosilicate Glass	PMMA	PDMS	Rogers (RO4000 Series)	LTCC
Relative permittivity (-)	10–11	3.8–5.1	3.2	2.3–2.8	3.3–3.5	7.5–7.8
Dielectric loss tangent (-)	0.001 @ 1MHz	0.002–0.004	0.001–0.003 @ 8.92 GHz	0.02 @ 2.5 GHz	0.002–0.003	0.006 @ 3 GHz
Resistivity (Ω·cm)	6.4 × 10^4^	4 × 10^10^	2 × 10^15^	0.6 × 10^12^	n/n	>10^12^
Contact angle (DI water on nontreated surface)	~20–40	~70	~75	~90–110	n/n	~60–70

**Table 2:** The comparison between the silicon, borosilicate glass, polymethyl methacrylate (PMMA), PDMS, Rogers glass-epoxy laminates and LTCC. “+” represents good properties, “+/−” represents average or ambiguous properties, and “−” represents the weak ones.

Material	Silicon	Borosilicate Glass	PMMA	PDMS	Rogers Laminates	LTCC
Possibility of integrating and manufacturing microwave circuits’ components.	+	+	+/−	+/−	+	+
Possibility of manufacturing the microfluidic components.	+	+	+	+	−	+
Possibility of operating in conditions with high humidity.	+	+	+	+	+/−	+
Harsh chemical solution resistivity.	+	+	+/−	+/−	−	+
Possibility of reusing the systems.	+	+	−	−	−	+
Possibility of fast prototyping.	+/−	+/−	+	+	+	+/−
Nonspecialized technology process.	−	−	+/−	+/−	+	+/−
The cost of fabricating the microsystems.	−	−	+	+	+	+/−
Accuracy of fabricating the microfluidic components.	+	+	+	+	n/n	+/−
Accuracy of fabricating the microwave components.	+	+	+/−	+/−	+	+

## Data Availability

Not applicable.
